# Effects of a Combined Front-of-Pack Nutrition Label and Nutrition Education Intervention on Healthier Choices of Freshly Prepared Beverages: An Online Randomized Controlled Trial

**DOI:** 10.3390/foods15101684

**Published:** 2026-05-12

**Authors:** Ruijia Shi, Jiazhang Huang, Junmao Sun

**Affiliations:** 1 Institute of Food and Nutrition Development, Ministry of Agriculture and Rural Affairs, Beijing 100081, China; shiruijia@caas.cn; 2 Institute of Quality Standard and Testing Technology for Agro-Products of Agriculture and Rural Affairs, Beijing 100081, China

**Keywords:** front-of-pack nutrition label, nutrition education, freshly prepared beverages, purchase decisions, healthfulness evaluations

## Abstract

Freshly prepared beverages are widely consumed in China, yet limited nutrition disclosure may hinder healthier choices. This study evaluated whether front-of-pack (FOP) nutrition labels combined with nutrition education improved stated purchase decisions and healthfulness evaluations for freshly prepared beverages beyond labels alone. Participants (*n* = 1100) were assigned to a combined intervention group (*n* = 551) or label-only group (*n* = 549) and completed six paired beverage tasks before and after intervention. Analyses used continuity-corrected McNemar tests, between-group net effects, transition analyses, and generalized estimating equations. For purchase decisions, healthier choices increased by 7.62–21.96 percentage points in the combined group versus 3.46–7.83 in the label-only group, with significant between-group net effects in five of six pairs after Holm correction. For healthfulness evaluations, improvements were 12.89–34.85 versus 6.19–9.29 percentage points, with significant between-group net effects in five of six pairs after Holm correction. Transition analyses showed larger shifts from non-healthier to healthier responses in the combined intervention group. Heterogeneity by sex and objective nutrition information literacy was significant only for healthfulness evaluations, with women and those with higher objective nutrition information literacy having additional benefit from the combined intervention. Therefore, adding nutrition education to FOP labels may promote healthier judgments and simulated choices for freshly prepared beverages.

## 1. Introduction

Freshly prepared beverages, characterized by diverse flavors, convenience, and strong social appeal, have become high-frequency items in the daily diets of Chinese residents, especially among young people and urban populations. Studies have shown that China’s freshly prepared beverage market has continued to expand in recent years, and that online ordering, instant delivery, and chain-brand development have further reinforced its high-frequency, convenient, and emotion-driven consumption characteristics [1,2]. However, rapid market growth has been accompanied by cumulative exposure to nutritional risks. Freshly prepared beverages are typically high in sugar, energy-dense, and highly variable in formulation. Compared with prepackaged foods, however, they still provide relatively limited nutrition information. As a result, consumers often find it difficult to identify product healthfulness accurately and in a timely manner, and may consume excessive added sugar without realizing it, thereby increasing the risk of overweight, obesity, and related chronic diseases [3,4]. Against the backdrop of the ongoing Healthy China strategy and increasingly stringent recommendations to control daily added sugar intake, exploring nutrition intervention tools suitable for freshly prepared beverage consumption has become an important issue in public nutrition and health promotion.

Nutrition labels are widely regarded as an important policy tool for improving diet quality and promoting healthier choices, but the practical role of traditional back-of-pack nutrition facts panels is often limited in real-world consumption settings. On the one hand, back-of-pack labels contain large amounts of information and require consumers to have a certain level of nutrition knowledge and invest time in interpretation. On the other hand, in fast-paced shopping and online ordering situations, consumers are more easily influenced by brand, taste, price, and promotional information, making it difficult for them to make full use of complex nutrition information [5]. To reduce the burden of information processing, the World Health Organization has recommended presenting nutrition information in a simpler, more intuitive, and more understandable format [6]. Front-of-pack (FOP) nutrition labeling has emerged as an important tool in this context. Compared with traditional nutrition facts panels, FOP nutrition labels can rapidly communicate the overall nutritional quality of a product through graphics, symbols, or grades, helping consumers compare healthfulness and make choices within a limited decision time [7,8]. Existing studies have shown that interpretive and summary FOP nutrition labels can improve consumers’ understanding of product healthfulness and, in some experimental and real-world settings, improve the nutritional quality of food choices [9,10].

Nevertheless, the behavioral effects of FOP nutrition labels are not always stable or consistent. Some studies have found that FOP nutrition labels can improve health perceptions and choice tendencies, but their behavioral effects are usually moderate and are easily influenced by consumers’ nutrition literacy, educational attainment, sex, age, and health motivation [11,12]. In freshly prepared beverage settings in particular, consumers often have to choose among multiple similar products within a short period of time. Nutritional judgment, therefore, depends not only on whether information is visible, but also on whether it is understandable, credible, and usable. From a dual-process theory, FOP labels may function as rapid heuristic cues that help consumers make quick comparative judgments, whereas nutrition education may promote more deliberative processing by translating abstract nutrient values into concrete and easily interpretable information [13]. This reasoning is also consistent with information-processing research suggesting that nutrition information is more likely to influence decision making when it is salient, understandable, and easy to use at the point of choice [5]. In digital ordering environments, such support may additionally operate as a form of digital nudging by structuring attention toward health-relevant cues without restricting choice [14,15]. Therefore, combining FOP labels with interactive nutrition education may be more effective than labels alone in improving both healthfulness evaluations and healthier stated choices. Accordingly, combining FOP nutrition labels with nutrition education that enhances comprehension, deepens information processing, and strengthens health motivation may be an important direction for improving intervention effectiveness. In digital platform environments, interactive nutrition education can support the understanding and use of label information by increasing audience engagement through scenario simulation, personalized feedback, and real-time information prompts.

In China, FOP nutrition labeling for freshly prepared beverages is increasingly being explored in policy and practice; however, systematic empirical evidence is still lacking on the actual effects of such labels in freshly prepared beverage settings, especially regarding how the combination of labeling and nutrition education influences consumers’ healthfulness evaluations and purchase decisions. In recent years, Shanghai and other regions have begun piloting health warning labels for sugar-sweetened beverages and “Nutrition Choice” graded labels, providing an institutional basis for the application of FOP nutrition labeling in the freshly prepared beverage sector. Existing research, however, has focused mainly on prepackaged foods or single-label interventions, leaving the effectiveness of the combined strategy of FOP nutrition labels plus nutrition education in freshly prepared beverage settings insufficiently understood. In particular, it remains unclear whether this combined intervention can further improve consumers’ purchase decisions and healthfulness evaluations beyond the effect of labeling alone, and whether its additional effects differ across population subgroups.

Against this background, guided by dual-process theory, nudge theory and information-processing theory, the present study examined whether FOP nutrition labels combined with interactive nutrition education would improve consumers’ responses to freshly prepared beverages beyond the effect of FOP labels alone. Specifically, we hypothesized that the combined intervention would lead to healthier purchase decisions (H1) and more accurate healthfulness evaluations (H2) than the label-only intervention. We further explored whether the additional effect of the combined intervention differed across population subgroups (RQ1). The novelty of this study lies not only in focusing on freshly prepared beverages, a category with limited standardized nutrition disclosure, but also in testing whether interactive nutrition education provides an additional benefit beyond FOP labels alone.

## 2. Materials and Methods

### 2.1. Study Design and Participants

This study used a two-arm randomized controlled experimental design embedded in an online questionnaire survey, with consumers of freshly prepared beverages as the target population. The survey was conducted via the professional online survey platform Wenjuanxing and was designed to simulate the real-world consumption setting in China, where freshly prepared beverages are primarily purchased through online ordering. Participants were recruited from the Wenjuanxing respondent panel, which includes a large pool of registered members. Eligibility was determined through a screening procedure in which participants were first provided with a definition of freshly prepared beverages and then asked whether they consumed such beverages at least once per month; only those answering “yes” were eligible. The inclusion threshold of consuming freshly prepared beverages at least once per month was used to ensure that participants had at least basic familiarity with this purchasing context. To identify valid responses, participants also had to indicate that they did purchase freshly prepared beverages; respondents selecting “do not purchase” in the brand-related question were excluded from the valid sample. A small monetary incentive was provided after questionnaire completion. After eligibility screening, participants were assigned to the combined intervention group or the label-only group using the platform’s built-in random assignment function. Allocation occurred within the survey platform after screening and before the intervention interface was displayed, thereby removing investigator influence on group assignment. Because the intervention conditions differed visibly owing to the presence or absence of interactive nutrition education, formal blinding of participants to intervention assignment was not feasible. However, participants were not informed of the specific comparative study hypothesis or the existence of alternative study conditions before completing the questionnaire. All procedures were administered automatically online without face-to-face interaction between investigators and participants during allocation or outcome assessment. The questionnaire was administered page by page without allowing participants to go back, so as to reduce response bias caused by retrospective modification. In addition, the platform’s random item presentation functions were used to randomize the order of beverage scenarios and the left-right display positions of beverages within each pair, so as to minimize order effects. Because the study relied on hypothetical choice tasks in a simulated online ordering context, the purchase outcome should be interpreted as a stated purchase choice rather than observed real-world purchasing behavior. After eligibility screening, 1100 valid samples were included, of which 551 were assigned to the combined intervention group and 549 to the label-only group.

### 2.2. Intervention Measures

**Figure 1:**
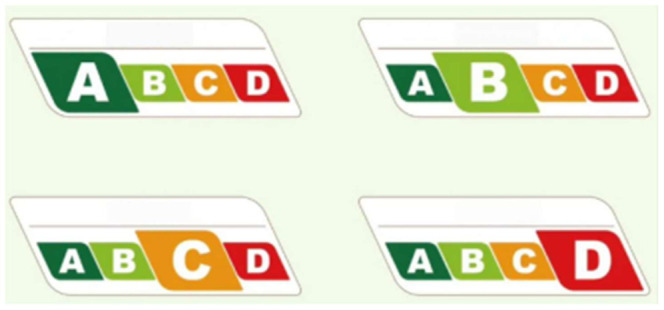
The “Nutrition Choice” graded label.

Two intervention groups were established in this study. The combined intervention group received both FOP nutrition labels and interactive nutrition education, whereas the label-only group received only the FOP nutrition label intervention. The FOP nutrition label used in this study was the “Nutrition Choice” graded label (Figure 1), which summarizes beverage nutritional quality using letter grades from A to D, with lower recommendation levels as the grade declines. The label has both color-coded and interpretive features, enabling participants to compare the relative nutritional quality of different beverages within a short time.

**Figure 2:**
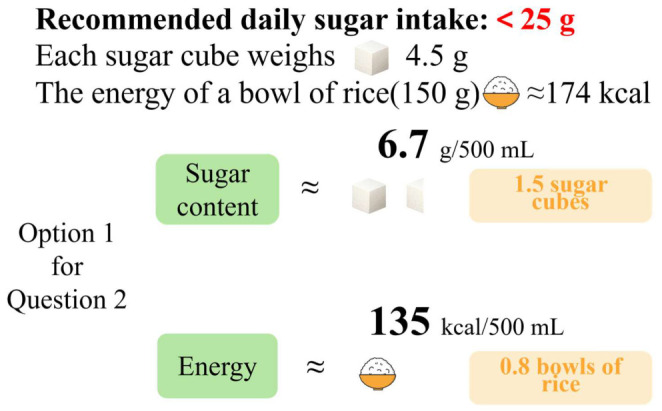
Interactive nutrition education (Using Option 1 for Question 2 as an example).

Interactive nutrition education (Figure 2, Appendix B) was delivered in real time during the choice tasks. When participants in the combined intervention group viewed or selected a beverage, the system displayed supplementary nutrition information specific to that beverage, presenting sugar content and energy in a more concrete and intuitive way, such as converting them into the equivalent number of sugar cubes and bowls of rice. This was intended to enhance participants’ understanding and perception of otherwise abstract nutrition information. If participants switched to another beverage option, the educational information changed accordingly. In the label-only group, the same choice interface displayed only the FOP nutrition label and did not provide such interactive educational information. Both groups completed the same paired beverage choice tasks and follow-up measurements, and the only difference between them was whether interactive nutrition education was provided.

The intervention was standardized in format across participants, while the specific numeric content varied according to the nutrient profile of the selected beverage. Participants could not bypass the choice task without selecting an option, the questionnaire was self-paced rather than time-fixed. The researchers recorded the time taken to complete the questionnaire. Before the main survey, pilot testing was conducted to assess questionnaire flow, intervention presentation, completion time, and the plausibility of response-quality screening rules. Based on the results of a pilot testing, conscientiously and fully completing the entire questionnaire typically requires approximately 10 min. Consequently, any responses completed in an unreasonably short amount of time were deemed invalid data and excluded. The intervention content was reviewed by two experts in the field of food nutrition economics and policy before formal data collection.

### 2.3. Questionnaire Structure and Measures

The questionnaire consisted of five parts. The first part collected baseline information on participants’ characteristics, including demographic characteristics, subjective nutrition information literacy, and objective nutrition information literacy. The second part assessed baseline consumption habits and perceptions, mainly measuring participants’ consumption frequency, familiarity, brand preferences, and related perceptions regarding freshly prepared beverages. The third part consisted of a choice experiment without intervention and was used to measure participants’ baseline purchase decisions and healthfulness evaluations. The fourth part consisted of a post-intervention choice experiment and was used to evaluate the effects of FOP nutrition labels and the combined intervention on purchase decisions and healthfulness evaluations. The fifth part contained post-intervention measures, mainly collecting participants’ evaluations of the FOP nutrition label and nutrition education use, including processing fluency, credibility, and liking.

To approximate real purchasing situations as closely as possible, the questionnaire measured purchase decisions before healthfulness evaluations at each stage so as to reduce the influence of health priming on food choice tasks. Six paired freshly prepared beverage scenarios were constructed, each containing two products with different nutritional profiles. Participants first answered the purchase decision question, “Which product would you buy?”, and then answered the healthfulness evaluation question, “Which product do you think is healthier?” To avoid order bias, the presentation order and left-right positions of the two beverages in each of the six scenarios were randomized. Participants could enlarge the images to view product information. During the intervention stage, locally enlarged FOP nutrition labels were displayed on the product interface to help participants identify label grades. The profiles of the 12 freshly prepared beverages are shown in Table 1.

The beverages were based on real products from brands involved in the Shanghai pilot context. To reduce explicit brand-name effects in the questionnaire, product names were not used in the task wording and options were anonymized as “Option 1” and “Option 2” within each question. However, real product images were retained to preserve ecological realism, and residual brand cues therefore cannot be completely excluded.

### 2.4. Main Study Variables

The main outcome variables in this study were purchase decisions and healthfulness evaluations. Purchase decision referred to the participant’s choice of which beverage to buy in each scenario, whereas healthfulness evaluation referred to the participant’s judgment of which beverage in each pair was relatively healthier (Appendix A and Appendix B). Because participants responded in hypothetical beverage-pair scenarios without actual payment or consumption, purchase decision was operationalized as a stated purchase choice in a simulated ordering task. The healthfulness evaluation item was used as a task-specific experimental outcome reflecting comparative perception within each beverage pair. This approach was conceptually adapted from experimental front-of-pack labeling studies that assess perceived healthfulness or judgments of product healthiness at the product level [16,17,18,19]. For pre-post comparison analyses, responses to each question were further dichotomized as a healthier choice (or a correct healthfulness evaluation) consistent with the direction of the FOP label grades versus a non-healthier choice (or non-healthier evaluation). This coding was used to align the analysis with the intervention objective, namely whether participants moved toward the option more consistent with the direction of the FOP label grades, and to facilitate within-participant pre-post comparison across beverage-pair scenarios. The healthier option was determined according to the FOP nutrition label grades, with A better than B, B better than C, and C better than D, and was coded based on the specific beverage pair.

In addition to the main outcomes, demographic variables including sex, age, place of residence, educational attainment, monthly household income, marital status, occupation, and body mass index (BMI), as well as objective nutrition information literacy scores (Appendix C), were collected to describe sample characteristics and conduct subgroup heterogeneity analyses.

### 2.5. Statistical Analysis

The combined strategy tested in this study—FOP label plus interactive nutrition education—was exploratory, and there was limited prior evidence on the expected additional effect size beyond a label-only intervention. Therefore, the final sample size was determined on the basis of feasibility and with reference to prior online front-of-pack labeling experiments, which have used sample sizes ranging from several hundred to several thousand participants depending on the number of study arms and outcomes assessed [20,21,22,23]. As a conservative achieved-sample benchmark, using a standard two-sample approximation for independent binary outcomes and ignoring efficiency gains from repeated within-participant observations, the achieved group sizes of 551 and 549 would be sufficient to detect an absolute between-group difference of approximately 8–9 percentage points with 80% power at a two-sided alpha of 0.05, using a conventional benchmark for statistical planning [24,25,26].

Descriptive statistics were first used to summarize demographic characteristics and baseline-related variables, with categorical variables presented as frequencies and percentages. At baseline, the distributions of purchase decisions and healthfulness evaluations across the six beverage-pair scenarios were compared for both the total sample and by study group, and chi-square tests based on contingency tables were used to assess between-group differences and determine baseline comparability after randomization.

For intervention effect analyses, comparisons were conducted at both the within-group and between-group levels. Within-group comparisons used continuity-corrected McNemar tests to examine whether the proportion of healthier choices or correct healthfulness evaluations changed significantly before and after the intervention for the same participant. For binary outcomes, effect sizes were expressed as absolute percentage-point changes from pre-intervention to post-intervention (Δpp) and as between-group differences in change (ΔΔ), both with 95% confidence intervals, because absolute percentage-point differences provide a directly interpretable estimate of intervention magnitude in public health and policy terms. In addition to the dichotomous pre-post comparison, individuals’ pre-post transitions were classified into four categories, namely “improved,” “worsened,” “remained non-healthier,” and “remained healthier,” and 2 × 4 chi-square tests were used to compare transition distributions between groups. Because multiple parallel comparisons were conducted for outcomes of the same type, the Holm–Bonferroni method was used to adjust for multiple testing and control the risk of type I error.

In subgroup heterogeneity analyses, repeated binary outcomes across beverage-pair scenarios were pooled and modeled using generalized estimating equation (GEE) logistic models with an exchangeable working correlation structure. This approach was used to account for within-participant correlation because each participant contributed multiple repeated responses across scenarios. The exchangeable structure assumes a common within-participant correlation across repeated observations and was considered appropriate because each participant contributed a fixed number of conceptually similar binary responses. By including three-way interaction terms of time × group × subgroup variable, the study tested whether the additional effect of the combined intervention relative to the label-only intervention differed by sex, age, educational attainment, monthly household income, BMI group, and objective nutrition information literacy, respectively. GEE was chosen because the primary interest was in estimating population-averaged intervention effects rather than participant-specific effects. Model diagnostics focused on model convergence and the stability of coefficient estimates. All exchangeable GEE models converged, with within-participant correlation estimates of 0.219–0.224 for healthfulness evaluations and 0.194–0.196 for purchase decisions. Taken together, these findings support the adequacy of the exchangeable GEE specification for the present repeated-measures data. All tests were two-sided, and statistical significance was defined as *p* < 0.05. Statistical analyses were all completed in Python 3.8.

## 3. Results and Discussion

### 3.1. Sample Characteristics

A total of 1100 valid samples were included in this study, including 551 in the combined intervention group and 549 in the label-only group. The overall sample showed a clear profile of core consumers of freshly prepared beverages (Table 2): women accounted for 60.2%, participants aged 25–34 years accounted for 56.5%, 93.7% lived in urban areas, 88.0% had a bachelor’s degree or above, monthly household income was concentrated mainly in the CNY 10,000–30,000 range (74.6%), and most participants were company employees (81.0%). The majority were married and had a normal BMI (76.4% and 71.5%, respectively). This structure indicates that the sample was not a random representation of the general population in China, but was more concentrated among high-frequency consumers of freshly prepared beverages and therefore better matched real consumption settings.

This result is broadly consistent with existing research and market observations on the profile of freshly prepared beverage consumers. Previous studies have shown that consumption of freshly prepared beverages in China is strongly characterized by youthfulness and urbanization, and that online ordering, instant delivery, and brand chain expansion have further reinforced the central role of young people and urban residents in such consumption [1]. Market research has also shown that the average age of consumers of freshly prepared beverages is about 25.5 years, and that white-collar workers and students are the main consumer groups, together accounting for more than 70% [27]. In the present study, the 25–34-year age group accounted for the largest share compared with other age groups, and company employees made up the overwhelming majority across occupations, followed by students, indicating that the sample structure is broadly aligned with the main consumer groups identified in previous studies. At the same time, the proportion of women exceeded that of men, which is also consistent with real-world observations that women are more sensitive to beverage consumption, flavor experimentation, and health information, suggesting that women may be both a key consumer group for freshly prepared beverages and an important target population for nutrition information interventions.

From the perspective of study fit, although this sample structure limits the direct generalizability of the findings to the national general population, it is well suited to evaluating the role of FOP nutrition labels and nutrition education in freshly prepared beverage settings. Consumption of freshly prepared beverages is not an isolated behavior detached from lifestyle, but is more deeply embedded in the everyday work, social, and leisure contexts of urban young adults. Therefore, a sample dominated by young, urban, highly educated, and middle- to upper-income participants not only more realistically reflects the core consumer profile of the current freshly prepared beverage market, but is also more conducive to testing the practical effects of nutrition information interventions in the actual target population. Overall, although the sample does not represent the national population structure, it is consistent with prior findings on the main consumer groups of freshly prepared beverages and thus possesses reasonable contextual external validity. While generalization to rural residents, older adults, and lower-literacy groups should still be made with caution.

### 3.2. Characteristics of Consumers’ Purchase Decisions and Healthfulness Evaluations Before the Intervention

Regarding baseline comparability between groups, no statistically significant differences were found between the combined intervention group and the label-only group in either purchase decisions or healthfulness evaluations across the six paired beverage choices (Table 3 and Table 4, all *p* > 0.05). This indicates that the two groups were well balanced before entering the intervention, and that the between-group differences observed subsequently are more likely to reflect the effects of the intervention itself rather than imbalances in baseline preferences.

Under conditions without any intervention, participants’ purchase decisions already showed some intuitive preferences. In the total sample, beverages such as Option 1 of Question 2, Option 2 of Question 5, and Option 2 of Question 6 were selected relatively more often, whereas the choice proportions for some beverage pairs were relatively close (Table 3). This suggests that in the absence of explicit nutritional information cues, consumers rely more on taste associations, product familiarity, and immediate appeal than on nutritional attributes when making choices. This finding is generally consistent with previous research on food and beverage consumption behavior, which shows that freshly prepared beverage consumption is strongly hedonic and contextual, with taste experience, convenience, and product image often carrying greater weight in short-term decisions [28,29].

In contrast, consumers’ judgments of beverage healthfulness showed a clearer directional pattern. In the total sample, beverages such as Option 2 of Question 5, Option 1 of Question 2, and Option 2 of Question 3, were more often rated as healthier (Table 4), indicating that even in the absence of FOP nutrition labels, consumers were still able to make an initial distinction regarding healthfulness based on product appearances, prior experience, or general common sense. This is consistent with previous findings that consumers can still form rough health judgments without labels; that is, consumers are not completely incapable of identifying healthfulness, but such judgments often remain at an experiential and heuristic level rather than being based on systematic nutritional information [30,31].

Further comparison of purchase decisions and healthfulness evaluations shows that what consumers “believe is healthier” does not fully align with what they are “actually more willing to buy.” For example, in the “Option 1 of Question 1/ Option 2 of Question 1” pair and the “Option 1 of Question 6/ Option 2 of Question 6” pair, participants tended to favor the former in healthfulness evaluations but preferred the latter in purchase decisions. This indicates that at baseline, consumers were not lacking a basic ability to recognize healthfulness, but rather that such health cognition had not yet been stably translated into actual choice behavior. This finding is consistent with previous research showing a disconnect between “health cognition” and “actual purchasing” in food choice, namely that when taste preference, pleasure, and habitual consumption are more salient, health attributes do not necessarily enter the core weighting of actual decisions automatically [10,28]. Thus, the baseline results of this study not only reveal a cognition-behavior gap in freshly prepared beverage consumption but also provide a realistic foundation for subsequently testing whether FOP nutrition labels and nutrition education can help bridge this gap.

### 3.3. Effects of the Combined Intervention on Purchase Decisions

**Figure 3:**
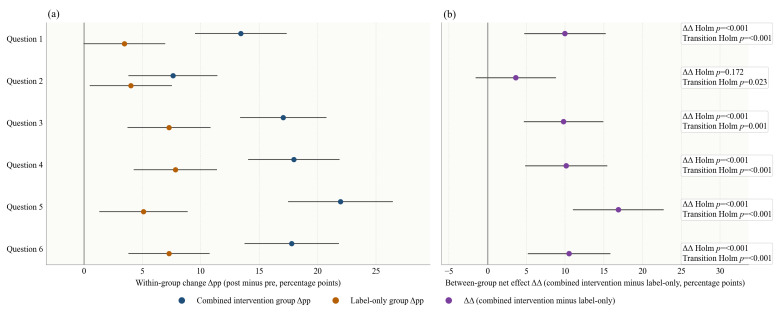
Forest plot of changes in participants’ purchase decisions before and after the FOP nutrition label and nutrition education intervention. (**a**) Within-group change effects (Δpp, i.e., the percentage-point change in the proportion of healthier purchase decisions after versus before the intervention) and their 95% CIs for the combined intervention group and the label-only group across beverage pairs. (**b**) Between-group net effect (ΔΔ, i.e., Δpp in the combined intervention group minus Δpp in the label-only group) and its 95% CIs.

Figure 3 and Table 5 show that FOP nutrition labels combined with nutrition education improved participants’ purchase decisions more than the label-only intervention in most beverage-pair scenarios. Across all six pairs, the combined intervention group showed larger increases in healthier choices than the label-only group, and the between-group net effect (ΔΔ) was statistically significant in five of the six comparisons after Holm adjustment. The largest additional effect was observed for Question 5 (ΔΔ = 16.86 percentage points, 95% CI: 11.00 to 22.72).

This result is broadly consistent with existing research on the effectiveness of FOP nutrition labels. Previous studies have shown that highly interpretive FOP nutrition labels can improve consumers’ ability to identify healthier foods and, to some extent, promote healthier choices [10,32,33]. The results obtained in the freshly prepared beverage setting further indicate that even in beverage purchasing environments characterized by fast ordering, strong sensory appeal, and more intuition-driven choices, label information can still guide behavior. At the same time, the present study also confirms the view in the literature that “improving cognition is easier than changing behavior.” Although the label-only group also showed some improvement, the overall increase was limited, suggesting that labels alone may still be insufficient to consistently move consumers from “understanding information” to “changing choices.” This is in line with the findings of Courbet et al. [34], who argued that FOP nutrition labels are more likely to affect cognitive evaluation and purchase intention first, and only then gradually influence behavior. A possible explanation is that the combined intervention influenced not only whether nutrition information was visible, but also how easily it could be cognitively processed and applied during choice. From a dual-process theory, FOP labels may function as rapid heuristic cues, whereas interactive nutrition education may encourage more deliberative processing by translating abstract nutrient values into concrete and easily interpretable units [13]. This interpretation is also consistent with nudge theory and information-processing theory, which suggest that nutrition information is more likely to influence choice when it is salient, timely, and easy to use at the point of decision, especially in online environments [5,14].

Compared with studies examining only the effect of labels alone, the value of this study lies more in demonstrating the additional benefit of the combined intervention. Previous research has found that static explanatory materials or supplementary reference information do not always produce stable improvements in purchasing behavior, and that the combined effects of labels and auxiliary information vary across settings [35]. In the present study, interactive nutrition education amplified the behavioral effect of labels across multiple paired beverage choices, suggesting that real-time, concrete informational support may help consumers process nutrition information more effectively than general explanatory materials. In practical terms, the largest significant net effect on stated purchase decisions (ΔΔ = 16.86 percentage points) means that, per 100 participants, about 17 additional participants selected the healthier beverage under the combined intervention than under the label-only condition. This suggests that when nutritional differences between beverages are more pronounced, or when educational information more directly reinforces these differences, the added benefit of the combined intervention becomes more substantial. This also echoes prior studies of real-time feedback interventions, which suggest that consumers are more likely to adjust their original choices when information is presented in a more understandable, comparable, and decision-timely manner [36].

Overall, the effect of the combined intervention on purchase decisions can be characterized as “consistent in direction but varying in magnitude.” Its additional effect was positive in most beverage pairs, but the size of the effect was not identical across all pairs. This suggests that the combined intervention does not exert a completely homogeneous effect across all consumption settings, but is more likely to work in situations where nutritional differences are larger and nutrition education information is easier to absorb. Accordingly, in freshly prepared beverage consumption, FOP nutrition labels can serve as an important informational cue for healthier choices, while the addition of nutrition education further increases the likelihood that this cue will be understood, compared, and translated into actual choices. In this way, healthier choices become more likely to move beyond cognition and be reflected in real purchase decision changes.

**Figure 4:**
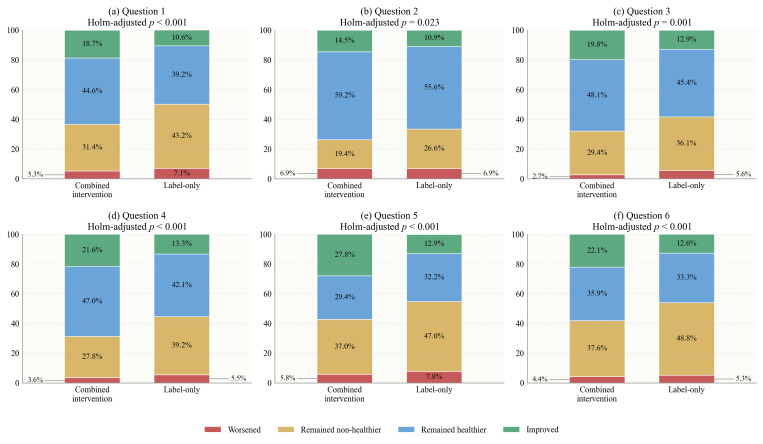
Individual-level transition distributions before and after the intervention for purchase decisions. Improved indicates a transition from a non-healthier choice/evaluation before the intervention to a healthier choice/evaluation after the intervention. Worsened indicates a transition from a healthier choice/evaluation before the intervention to a non-healthier choice/evaluation after the intervention. Remained non-healthier and remained healthier indicate unchanged response patterns before and after the intervention.

Δpp indicates the percentage-point change in the proportion of healthier purchase decisions after versus before the intervention; ΔΔ indicates the difference in Δpp between the combined intervention group and the label-only group. The individual-level transition analysis further showed that the additional effect of the combined intervention was driven mainly by a higher proportion of participants moving from non-healthier to healthier choices and a lower proportion remaining in the non-healthier category. This pattern was observed across all six beverage pairs (all adjusted *p* < 0.05), with the clearest contrast again seen for Question 5, where 27.8% of participants in the combined intervention group improved, compared with 12.9% in the label-only group, while the proportion remaining non-healthier was 37.0% versus 47.0% (Figure 4).

This transition pattern supports the interpretation that interactive nutrition education may have improved the usability of FOP label information. Previous studies have suggested that FOP labels can guide healthier choices, but their behavioral effects are often modest and context-dependent [10,32,33,37]. The present transition results show that the combination of labels and real-time educational cues helped a larger share of participants revise their initial choices in the healthier direction. This is consistent with evidence that label-plus interventions, warning labels, and timely cognitive aids can strengthen the translation of nutrition information into action, particularly when information is concrete, easy to compare, and delivered close to the moment of choice [31,36,38,39].

### 3.4. Effects of the Combined Intervention on Healthfulness Evaluations

**Figure 5:**
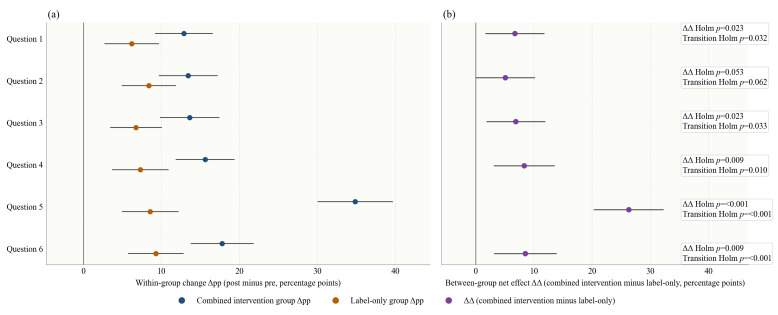
Forest plot of changes in participants’ healthfulness evaluations before and after the FOP nutrition label and nutrition education intervention. (**a**) Within-group change effects (Δpp, i.e., the percentage-point change in the proportion of healthier healthfulness evaluations after versus before the intervention) and their 95% CIs for the combined intervention group and the label-only group across beverage pairs. (**b**) Between-group net effect (ΔΔ, i.e., Δpp in the combined intervention group minus Δpp in the label-only group) and its 95% CIs.

Figure 5 and Table 6 show that both the combined intervention and the label-only intervention improved participants’ healthfulness evaluations, but the improvement was generally greater in the combined intervention group. The between-group net effect (ΔΔ) was statistically significant in five of the six beverage-pair scenarios after Holm adjustment, again with Question 5 showing the largest additional effect (ΔΔ = 26.28 percentage points, 95% CI: 20.26 to 32.31). Compared with purchase decisions, the effects on healthfulness evaluations were more widespread and more pronounced, suggesting that this outcome was more immediately responsive to nutrition information cues.

This pattern also aligns with the theoretical interpretation proposed above. From a dual-process theory, the interpretive FOP label may serve as a rapid cue for comparative health judgments, whereas purchase decisions are more likely to involve competing hedonic and habitual influences [13]. Similarly, from an information-processing theory, healthfulness evaluation requires less behavioral commitment than purchase choice and may therefore be more readily improved when nutrition information becomes salient and easy to interpret [37].

This finding is generally consistent with previous research. Existing studies have shown that FOP nutrition labels produce stronger and more stable effects in improving consumers’ understanding of product healthfulness than in changing actual purchasing behavior [10,34]. Similarly, the present study found that improvements in healthfulness evaluations were more widespread, suggesting that labels act first on cognitive judgment and only gradually influence behavioral choice. Compared with studies on prepackaged foods, the present study further demonstrates, in the context of freshly prepared beverages, that even in beverage environments where nutrition information disclosure is relatively limited and consumption decisions are made more quickly, FOP nutrition labels can still effectively enhance consumers’ ability to identify product healthfulness.

Further comparison of the magnitude of change between groups shows that the combined intervention still yielded obvious additional gains in most questions. As shown in Table 6, except for Question 2, the between-group net effect (ΔΔ) reached statistical significance in the other five questions, with the strongest additional effect observed for Question 5 at 26.28 percentage points. In practical terms, the largest net effect on healthfulness evaluations (ΔΔ = 26.28 percentage points) means that, per 100 participants, about 26 additional participants correctly identified the healthier option under the combined intervention than under the label-only condition. This indicates that nutrition education is not merely a repetitive supplement to label information, but in most situations further strengthens consumers’ ability to identify and interpret nutritional differences, making originally abstract nutritional grade information more easily translated into clear healthfulness judgments. This finding is consistent with research showing that interactive or interpretive supporting information can improve label comprehensibility: when consumers not only “see” the label but also understand its meaning more concretely with educational support, the cognitive effect of the label can be further amplified [40].

From the overall pattern, the between-group net effects for all six questions in Figure 5 were positive, indicating that the direction of effect of the combined intervention was better than that of the label-only intervention for every question. The differences lie mainly in magnitude rather than in opposite directions. This means that the combined intervention has relatively good generalizability in promoting healthfulness evaluations, and that the variation across questions more likely reflects differences in how recognizable nutritional differences are across beverage-pair scenarios. In particular, Question 5 showed the strongest effect, suggesting that when nutritional differences between beverages are larger, or when educational information more readily concretizes such differences, consumers are more likely to form consistent healthfulness evaluations. By contrast, although Question 2 also showed a positive trend, it did not reach statistical significance, suggesting that in some situations where baseline healthfulness evaluations are already relatively clear, labels alone may already provide a strong enough cue, thereby limiting the additional room for improvement created by the combined intervention.

Overall, the findings indicate that FOP nutrition labels themselves can effectively improve consumers’ judgments of the healthfulness of freshly prepared beverages, and that adding nutrition education further strengthens this cognitive improvement in most questions. When compared with the purchase decision results in the previous subsection, improvements in healthfulness evaluations were more widespread and more easily reached significance, even in the label-only group. This suggests that healthfulness evaluation may be the earlier, front-end stage through which the combined intervention first operates, whereas purchase decision is a behavioral outcome that, on the basis of cognitive improvement, is further shaped by taste preferences, consumption habits, and product appeal. Thus, the primary value of nutrition education lies not only in increasing the amount of information available, but in helping consumers understand and use label information more fully, thereby improving their ability to distinguish the healthfulness of freshly prepared beverages.

**Figure 6:**
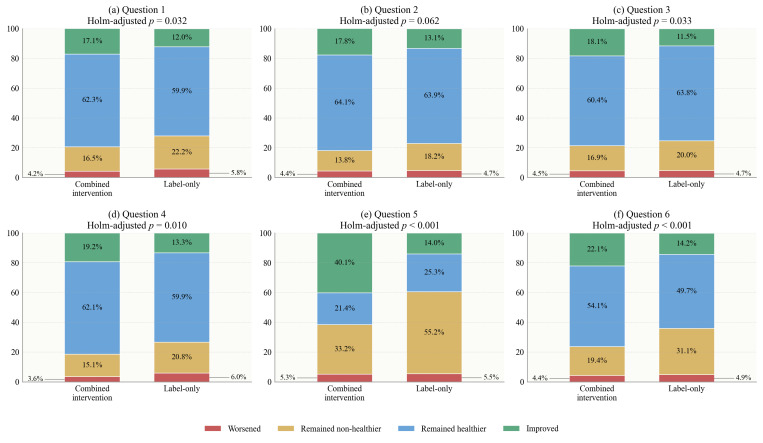
Individual-level transition distributions before and after the intervention for healthfulness evaluations. Improved indicates a transition from a non-healthier choice/evaluation before the intervention to a healthier choice/evaluation after the intervention. Worsened indicates a transition from a healthier choice/evaluation before the intervention to a non-healthier choice/evaluation after the intervention. Remained non-healthier and remained healthier indicate unchanged response patterns before and after the intervention.

Δpp indicates the percentage-point change in the proportion of healthier healthfulness evaluations after versus before the intervention; ΔΔ indicates the difference in Δpp between the combined intervention group and the label-only group. For healthfulness evaluations, the transition analysis showed a similar but more cognitively pronounced pattern (Figure 6). The combined intervention group consistently showed a larger proportion of improved responses and a smaller proportion remaining non-healthier than the label-only group. The largest difference again appeared in Question 5: 40.1% of participants in the combined intervention group improved, compared with 14.0% in the label-only group, whereas the proportion remaining non-healthier was substantially lower in the combined intervention group than in the label-only group (33.2% vs. 55.2%).

These findings complement the ΔΔ results by showing that the combined intervention changed not only the overall proportion of healthier evaluations, but also the distribution of individual response trajectories. The stronger transition effect for healthfulness evaluations than for purchase decisions suggests that nutrition education first operated through cognitive judgment, helping participants better interpret the meaning of FOP label grades and identify the healthier option. This interpretation is supported by experimental evidence showing that FOP labels improve objective understanding of nutritional quality and perceived healthfulness, including in beverage-specific settings [18,37].

### 3.5. Subgroup Heterogeneity Analysis

**Figure 7:**
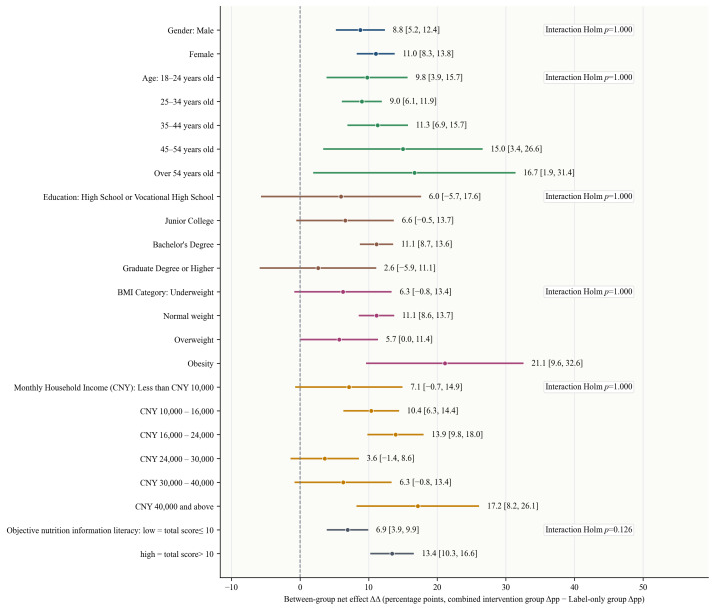
Forest plot of subgroup heterogeneity in the effect of the combined intervention on participants purchase decisions (pooled across six questions). Δpp represents the percentage-point change in the proportion of healthier purchase decisions from before to after the intervention. ΔΔ = Δpp in the combined intervention group − Δpp in the label-only group) and its 95% CIs.

For the outcome of purchase decision, subgroup analyses showed that none of the interaction effects for subgroup variables remained statistically significant after Holm adjustment, suggesting that the additional benefit of the combined intervention relative to the label-only intervention in promoting healthier purchase decisions did not show clear significant heterogeneity. However, Figure 7 shows that the between-group net effects were positive across all subgroup levels, indicating that the direction of the effect of the combined intervention was generally consistent across populations, with greater improvement than the label-only intervention in all cases. This suggests that the combined intervention has relatively good general applicability at the behavioral level, and that the differences across groups are more about the extent of improvement than a fundamental distinction in whether the intervention works at all. This result is broadly consistent with previous findings that FOP nutrition labels are often less likely to produce stable population stratification at the behavioral use level. Compared with cognitive judgments, actual choices are more susceptible to multiple non-nutritional factors, so the behavioral gains from labels and educational information are more likely to be universal but vary in magnitude rather than showing clear subgroup segmentation [41].

**Figure 8:**
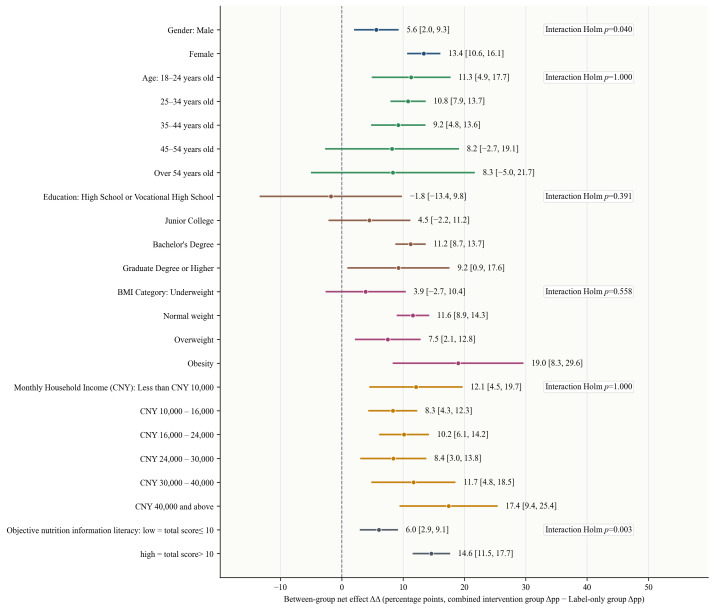
Forest plot of subgroup heterogeneity in the effect of the combined intervention on participants healthfulness evaluations (pooled across six questions). Δpp represents the percentage-point change in the proportion of healthier healthfulness evaluations from before to after the intervention. ΔΔ = Δpp in the combined intervention group − Δpp in the label-only group) and its 95% CIs.

By contrast, heterogeneity was clearer for the outcome of healthfulness evaluation. In terms of specific effects, the additional benefit of the combined intervention was clearly stronger among women than among men (Figure 8). This result is consistent with previous findings that women are more likely to attend to, understand, and use nutrition label information [42], and it also suggests that women may be more sensitive to health cues and risk prompts in freshly prepared beverage consumption. It may also reflect broader social and experiential differences, such as more frequent engagement with dietary information, body-weight management, or health-related food evaluation, which could make the educational content more immediately meaningful for some female participants. Similarly, the gain among individuals with higher objective nutrition information literacy was clearly greater than among those with lower literacy. This suggests that although nutrition education can generally strengthen the cognitive effect of labels, its effectiveness still depends to some extent on an individual’s existing nutrition knowledge base [43]. When participants possess better objective nutrition information comprehension, the combined intervention is more easily absorbed accurately and translated into clear healthfulness evaluations. This result is consistent with previous research indicating that label use is influenced by nutrition knowledge and information literacy, and further suggests that in products such as freshly prepared beverages, where health risks are not sufficiently intuitive, information-processing ability remains an important condition shaping intervention effectiveness [40,41].

Overall, the subgroup analyses suggest that the promoting effect of the combined intervention on purchase decisions is generally consistent across populations, with stable and widespread positive effects, whereas for healthfulness evaluations the additional benefit is more concentrated among women and those with higher objective nutrition information literacy. These differences may have implications for how nutrition interventions are designed and communicated. Optimizing modes of expression and strengthening interpretive support may further improve intervention effectiveness and equity of coverage.

### 3.6. Limitations

This study has several limitations. First, although the outcomes were derived from hypothetical choice tasks rather than observed purchases, the online format itself was closely aligned with the target consumption environment. In China, freshly prepared beverages are commonly purchased through app-based or other digital ordering interfaces; therefore, the present study did not attempt to simulate an unfamiliar setting, but rather to approximate a highly relevant real-world decision context [44]. Accordingly, the main limitation lies in the use of stated choices without actual payment or consumption, rather than in a strong mismatch between online and offline choice environments. Second, because the intervention conditions differed visibly, formal participant blinding to intervention assignment was not feasible in this online experiment, although participants were not informed of the specific comparative study hypothesis before task completion. Third, the study should be interpreted in light of its policy context, but its implications are not limited to China alone. The broader choice architecture examined here—rapid app-based ordering, reliance on visual product presentation, and limited nutrition transparency—is increasingly common in digital food environments internationally [44]. Consistent with this, online menu-labeling and FOP labeling experiments conducted in other countries have also shown that digital nutrition cues can influence product evaluations, knowledge, and hypothetical choices [18,39]. Thus, the findings may have broader conceptual relevance for similar digital beverage-ordering settings beyond China. Nevertheless, direct policy translation should still be made with caution, because countries differ in their FOP label systems, regulatory frameworks, and consumers’ familiarity with nutrition labeling.

## 4. Conclusions

This study has extended the existing literature in two important ways. First, it applied FOP nutrition labeling to freshly prepared beverages, a consumption setting that differs from prepackaged foods because products are often customized, purchased quickly, and accompanied by limited standardized nutrition disclosure. Second, rather than evaluating labels alone, this study tested a combined intervention that integrated FOP labels with real-time, beverage-specific nutrition education. The results suggest that nutrition education can improve the usability of label information by translating abstract nutrition indicators into more concrete and interpretable cues. Beyond increasing the overall proportion of healthier purchase decisions and healthfulness evaluations, the combined intervention also produced more favorable individual-level transitions from non-healthier to healthier responses, suggesting that nutrition education improved the usability of label information.

Accordingly, the focus of health interventions for freshly prepared beverages should not remain at the level of “providing information,” but should shift further toward “improving information usability.” These findings suggest that combining interpretive FOP labels with contextualized nutrition education may be useful in digital ordering interfaces, delivery-platform menus, and related online beverage-purchasing settings. Although the present study was conducted in China, the broader choice architecture examined here is not unique to China and may also be relevant to similar digital beverage-ordering settings in other countries. However, direct policy translation should still consider differences in local label systems, regulatory environments, and consumer familiarity with nutrition labeling.

## Figures and Tables

**Table 1:** Profiles of 12 freshly prepared beverages.

Order	Freshly Prepared Beverages	Sugar Content (g/500 mL)	Energy (kcal/500 mL)	“Nutrition Choice” Graded Label
Question 1	Option 1	1.2	0	A
	Option 2	12	243	D
Question 2	Option 1	6.7	135	B
	Option 2	10.6	211	C
Question 3	Option 1	7.3	145	C
	Option 2	5.2	105	B
Question 4	Option 1	1.2	0	A
	Option 2	8.5	170	B
Question 5	Option 1	17.2	344	B
	Option 2	29.4	587.31	D
Question 6	Option 1	1	11	A
	Option 2	5.8	115	B

**Table 2:** Basic characteristics of participants.

Variables	Items	Total Sample (*n* = 1100)	Combined Intervention Group (*n* = 551)	Label-Only Group (*n* = 549)
Gender	Male	438 (39.8%)	221 (40.1%)	217 (39.5%)
	Female	662 (60.2%)	330 (59.9%)	332 (60.5%)
Age	18–24 years old	140 (12.7%)	67 (12.2%)	73 (13.3%)
	25–34 years old	622 (56.5%)	321 (58.3%)	301 (54.8%)
	35–44 years old	284 (25.8%)	129 (23.4%)	155 (28.2%)
	45–54 years old	39 (3.5%)	24 (4.4%)	15 (2.7%)
	Over 54 years old	15 (1.4%)	10 (1.8%)	5 (0.9%)
Place of Permanent Residence	Rural	69 (6.3%)	35 (6.4%)	34 (6.2%)
	Urban	1031 (93.7%)	516 (93.6%)	515 (93.8%)
Education	High School or Vocational High School	31 (2.8%)	18 (3.3%)	13 (2.4%)
	Junior College	101 (9.2%)	41 (7.4%)	60 (10.9%)
	Bachelor’s Degree	876 (79.6%)	442 (80.2%)	434 (79.1%)
	Graduate Degree or Higher	92 (8.4%)	50 (9.1%)	42 (7.7%)
Monthly Household Income (CNY)	Less than CNY 10,000	89 (8.1%)	39 (7.1%)	50 (9.1%)
	CNY 10,000–16,000	285 (25.9%)	130 (23.6%)	155 (28.2%)
	CNY 16,000–24,000	310 (28.2%)	160 (29.0%)	150 (27.3%)
	CNY 24,000–30,000	225 (20.5%)	115 (20.9%)	110 (20.0%)
	CNY 30,000–40,000	114 (10.4%)	63 (11.4%)	51 (9.3%)
	CNY 40,000 and above	77 (7.0%)	44 (8.0%)	33 (6.0%)
Marriage	Single	260 (23.6%)	128 (23.2%)	132 (24.0%)
	Married	840 (76.4%)	423 (76.8%)	417 (76.0%)
BMI group	Underweight (BMI < 18.5)	117 (10.6%)	63 (11.4%)	54 (9.8%)
	Normal (18.5 ≤ BMI < 24)	786 (71.5%)	377 (68.4%)	409 (74.5%)
	Overweight (24 ≤ BMI < 27.9)	166 (15.1%)	96 (17.4%)	70 (12.8%)
	Obese (BMI ≥ 28)	31 (2.8%)	15 (2.7%)	16 (2.9%)
Occupation	Students	84 (7.6%)	37 (6.7%)	47 (8.6%)
	Civil Servants/Public Institution Staff	76 (6.9%)	41 (7.4%)	35 (6.4%)
	Corporate Employees	891 (81.0%)	447 (81.1%)	444 (80.9%)
	Self-Employed Individuals	30 (2.7%)	16 (2.9%)	14 (2.6%)
	Freelancers (Unemployed)	19 (1.7%)	10 (1.8%)	9 (1.6%)

**Table 3:** Participants’ purchase decisions before the front-of-pack nutrition label and nutrition education intervention.

Order	Items	Total Sample (*n* = 1100)	Combined Intervention Group (*n* = 551)	Label-Only Group (*n* = 549)	*p*
Question 1	Option 1	529 (48.1%)	275 (49.9%)	254 (46.3%)	0.227
	Option 2	571 (51.9%)	276 (50.1%)	295 (53.7%)	
Question 2	Option 1	707 (64.3%)	364 (66.1%)	343 (62.5%)	0.215
	Option 2	393 (35.7%)	187 (33.9%)	206 (37.5%)	
Question 3	Option 1	540 (49.1%)	271 (49.2%)	269 (49.0%)	0.951
	Option 2	560 (50.9%)	280 (50.8%)	280 (51.0%)	
Question 4	Option 1	540 (49.1%)	279 (50.6%)	261 (47.5%)	0.305
	Option 2	560 (50.9%)	272 (49.4%)	288 (52.5%)	
Question 5	Option 1	414 (37.6%)	194 (35.2%)	220 (40.1%)	0.096
	Option 2	686 (62.4%)	357 (64.8%)	329 (59.9%)	
Question 6	Option 1	434 (39.5%)	222 (40.3%)	212 (38.6%)	0.570
	Option 2	666 (60.5%)	329 (59.7%)	337 (61.4%)	

**Table 4:** Participants’ healthfulness evaluations before the front-of-pack nutrition label and nutrition education intervention.

Order	Items	Total Sample (*n* = 1100)	Combined Intervention Group (*n* = 551)	Label-Only Group (*n* = 549)	*p*
Question 1	Option 1	727 (66.1%)	366 (66.4%)	361 (65.8%)	0.815
	Option 2	373 (33.9%)	185 (33.6%)	188 (34.2%)	
Question 2	Option 1	754 (68.5%)	377 (68.4%)	377 (68.7%)	0.929
	Option 2	346 (31.5%)	174 (31.6%)	172 (31.3%)	
Question 3	Option 1	366 (33.3%)	193 (35.0%)	173 (31.5%)	0.216
	Option 2	734 (66.7%)	358 (65.0%)	376 (68.5%)	
Question 4	Option 1	724 (65.8%)	362 (65.7%)	362 (65.9%)	0.933
	Option 2	376 (34.2%)	189 (34.3%)	187 (34.1%)	
Question 5	Option 1	316 (28.7%)	147 (26.7%)	169 (30.8%)	0.133
	Option 2	784 (71.3%)	404 (73.3%)	380 (69.2%)	
Question 6	Option 1	622 (56.5%)	322 (58.4%)	300 (54.6%)	0.204
	Option 2	478 (43.5%)	229 (41.6%)	249 (45.4%)	

**Table 5:** Participants’ purchase decisions before and after the FOP nutrition label and nutrition education intervention.

Order	Items	Better Option	Combined Intervention Group			Label-Only Group			Between-Group Net Effect ΔΔ	ΔΔ Holm-Adjusted *p* Value
			Number (Percentage) of Better Options Pre-Intervention	Number (Percentage) of Better Options Post-Intervention	Δpp (95% CI)	Number (Percentage) of Better Options Pre-Intervention	Number (Percentage) of Better Options Post-Intervention	Δpp (95% CI)		
Question 1	Option 1	Option 1	275 (49.9)	349 (63.3)	13.43 (9.50, 17.36)	254 (46.3)	273 (49.7)	3.46 (−0.04, 6.97)	9.97 (4.70, 15.23)	<0.001
	Option 2									
Question 2	Option 1	Option 1	364 (66.1)	406 (73.7)	7.62 (3.81, 11.43)	343 (62.5)	365 (66.5)	4.01 (0.49, 7.53)	3.62 (−1.57, 8.80)	0.172
	Option 2									
Question 3	Option 1	Option 2	280 (50.8)	374 (67.9)	17.06 (13.36, 20.76)	280 (51.0)	320 (58.3)	7.29 (3.73, 10.84)	9.77 (4.65, 14.90)	<0.001
	Option 2									
Question 4	Option 1	Option 1	279 (50.6)	378 (68.6)	17.97 (14.05, 21.88)	261 (47.5)	304 (55.4)	7.83 (4.27, 11.40)	10.13 (4.84, 15.43)	<0.001
	Option 2									
Question 5	Option 1	Option 1	194 (35.2)	315 (57.2)	21.96 (17.48, 26.44)	220 (40.1)	248 (45.2)	5.10 (1.31, 8.89)	16.86 (11.00, 22.72)	<0.001
	Option 2									
Question 6	Option 1	Option 1	222 (40.3)	320 (58.1)	17.79 (13.75, 21.82)	212 (38.6)	252 (45.9)	7.29 (3.80, 10.77)	10.50 (5.17, 15.83)	<0.001
	Option 2									

**Table 6:** Participants’ healthfulness evaluations before and after the FOP nutrition label and nutrition education intervention.

Order	Items	Better Option	Combined Intervention Group			Label-Only Group			Between-Group Net Effect ΔΔ	ΔΔ Holm-Adjusted *p* Value
			Number (Percentage) of Better Options Pre-Intervention	Number (Percentage) of Better Options Post-Intervention	Δpp (95% CI)	Number (Percentage) of Better Options Pre-Intervention	Number (Percentage) of Better Options Post-Intervention	Δpp (95% CI)		
Question 1	Option 1	Option 1	366 (66.4%)	437 (79.3%)	12.89 (9.19, 16.58)	361 (65.8)	395 (71.9)	6.19 (2.70, 9.69)	6.69 (1.61, 11.78)	0.023
	Option 2									
Question 2	Option 1	Option 1	377 (68.4%)	451 (81.9%)	13.43 (9.66, 17.20)	377 (68.7)	423 (77.0)	8.38 (4.91, 11.84)	5.05 (−0.07, 10.17)	0.053
	Option 2									
Question 3	Option 1	Option 2	358 (65.0%)	433 (78.6%)	13.61 (9.80, 17.42)	376 (68.5)	413 (75.2)	6.74 (3.42, 10.06)	6.87 (1.82, 11.93)	0.023
	Option 2									
Question 4	Option 1	Option 1	362 (65.7%)	448 (81.3%)	15.61 (11.83, 19.38)	362 (65.9)	402 (73.2)	7.29 (3.66, 10.91)	8.32 (3.09, 13.55)	0.009
	Option 2									
Question 5	Option 1	Option 1	147 (26.7%)	339 (61.5%)	34.85 (30.03, 39.66)	169 (30.8)	216 (39.3)	8.56 (4.94, 12.18)	26.28 (20.26, 32.31)	<0.001
	Option 2									
Question 6	Option 1	Option 1	322 (58.4%)	420 (76.2%)	17.79 (13.75, 21.82)	300 (54.6)	351 (63.9)	9.29 (5.71, 12.86)	8.50 (3.11, 13.89)	0.009
	Option 2									

**Figure A1:**
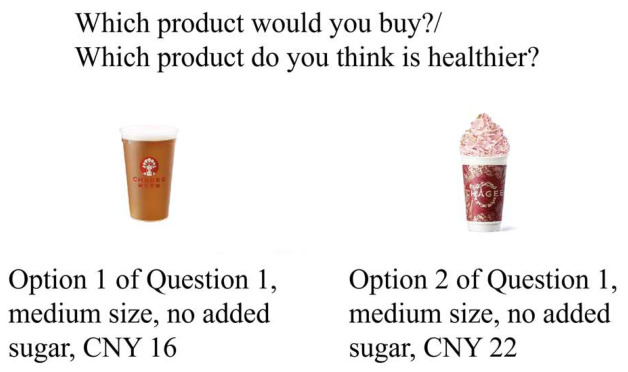
Example of the purchase decision and healthfulness evaluation tasks.

**Figure A2:**
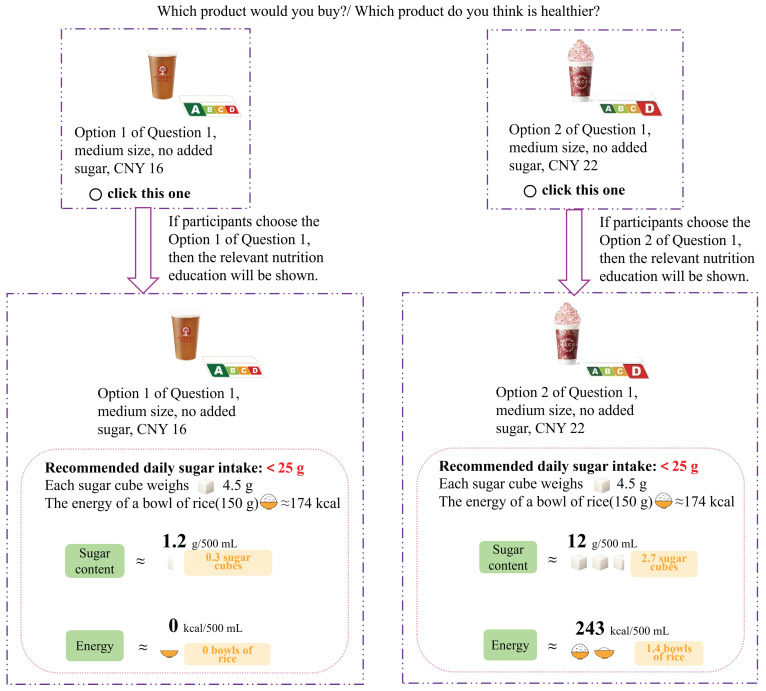
Example of the combined intervention group.

**Figure A3:**
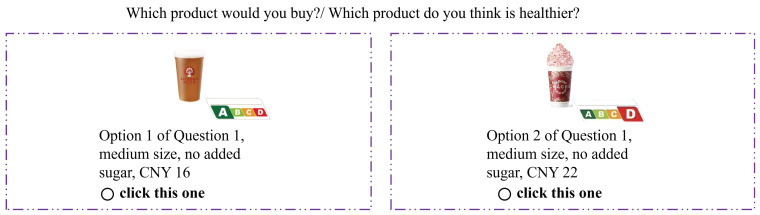
Example of the label-only group.

## Data Availability

The data presented in this study are available on request from the corresponding authors. The data are not publicly available due to privacy restrictions.

## Appendix A

Appendix A presents baseline measurements for participants’ purchase decisions and healthfulness evaluations. To avoid excessively priming participants to focus on health factors in food choice, this study first measured consumers’ purchase decisions and then their healthfulness evaluations. First, six pairs of freshly prepared beverages were presented sequentially in random order, with each pair containing two products that differed in healthfulness and displayed no FOP nutrition label information. Figure A1 presents an example of the purchase decision and healthfulness evaluation tasks. Participants were asked to select which of the two displayed products they would purchase. After completing all purchase decision tasks, the six beverage pairs were presented again in random order, and participants were asked to choose which product in each pair they considered healthier. To avoid order effects, the left-right positions of the two products within each beverage pair were randomized. Participants could enlarge the product images to view the front-of-package information.

## Appendix B

After the baseline measurement, participants were randomly assigned to one of two intervention groups: the combined intervention group or the label-only group. Figure A2 presents an example of the combined intervention group. In this group, the freshly prepared beverage choice interface contained FOP nutrition label information, and when participants selected a beverage, a real-time interactive nutrition education panel tailored to that specific beverage appeared. Figure A3 presents an example of the label-only group. In this group, the beverage choice interface included only the FOP nutrition label intervention, without tailored interactive nutrition education. Participants in both groups completed the same purchase decision and healthfulness evaluation tasks as in the baseline measurement. As in the pre-intervention task, the order of the two products within each beverage pair was randomized. Clicking on the product image enlarged it for closer viewing, and the FOP nutrition label on the product was also partially enlarged to facilitate careful inspection by participants.

## Appendix C

Objective nutrition information literacy in this study was operationalized as a content-based knowledge index to capture objective knowledge rather than as a reflective psychometric scale. The content was reviewed by experts before formal data collection. Objective nutrition information literacy was measured using three single-choice questions and four multiple-choice questions. A correct answer on a single-choice question scored 1 point; otherwise, 0 points were assigned. For multiple-choice questions, each correctly selected option scored 1 point, but selecting any incorrect option resulted in a score of 0 for that item. The total score was 21 points (Table A1).

**Table A1:** Scale for measuring objective nutrition information literacy.

Category	Measurement Item	Options (Correct Answers in Bold)
Single-choice	Do you know the recommended maximum daily sugar intake?	**No more than 50 g per day**, No more than 60 g per day, No more than 70 g per day, No more than 80 g per day, Don’t know
	Do you know the recommended ideal daily sugar intake?	About 20 g per day, **About 25 g per day**, About 30 g per day, About 35 g per day, Don’t know
	Do you know the World Health Organization’s acceptable daily intake of non-sugar sweeteners?	20 mg/kg body weight, **40 mg/kg body weight**, 60 mg/kg body weight, 80 mg/kg body weight, Don’t know
Multiple-choice	Do you know what problems excessive sugar intake may cause?	**Obesity**, **Dental caries**, **Diabetes**, **Cardiovascular disease**, Don’t know
	Do you know which of the following are non-milk-derived sugars? Note: non-milk-derived sugars refer to added sugars other than lactose.	**White sugar**, **Fructose**, **Honey**, **Fruit juice**, **Syrup**, Don’t know
	Do you know which of the following are artificial sweeteners?	**Aspartame**, **Acesulfame potassium**, **Neotame**, **Saccharin**, Stevia, **Sucralose**, Mogroside, Glycyrrhizin, Erythritol, Don’t know
	Do you know which of the following are natural sugar substitutes?	Aspartame, Acesulfame potassium, Neotame, Saccharin, **Stevia**, Sucralose, **Mogroside**, **Glycyrrhizin**, **Erythritol**, Don’t know
